# Practical Dentistry

**Published:** 1878-04

**Authors:** W. C. Duncan

**Affiliations:** Cincinnati


### PRACTICAL DENTISTRY.

BY W. C. DUNCAN, D. D. S. CINCINNATI.

SYNONYMS—Mechanical; Operative; Manual.

Dentistry is a branch of that science and art which has for its object the prevention and treatment of disease; having for its province the teeth and their appendages, and the construction and insertion of artificial teeth, when the natural organs are lost; and can appropriately be divided into medical and practical dentistry.

The correlation of forces in dentistry are such that no one can be ignored without doing irreparable injury to the whole. In proportion as these forces are evolved by the action of the mind, properly understood and acted on, the more beauty and harmony will reveal itself, and in a corresponding degree will the dentist be truly elevated in his own estimation and in that of his fellow men. Such a man will appear in a very marked contrast to those asses in the business who put on the lion's skin and if they did not attempt to roar might deceive. But they are so tickled by the assumption that they begin to bray.

The position taken by men of little mind, that the preparation of an artificial set (denture not in the English language) of teeth is low and menial and the only mechanical part of dentistry is a great mistake, showing a gross perversion of language. Very few men of this class have the ability to make a respectable piece of work. But they are mercenary enough to divide the money profit with what they term the mechanic if he will stultify himself enough to suffer it. A noted dental college professor who was visiting a dental society in Illinois was called upon for his views as to mechanical dentistry replied "I know nothing about it." Verily he told the truth. The time was when surgery was styled by many the mechanical part of medicine (Sir Astley Cooper a mechanic) and rejected from the Universities, and given over to barbers and montebanks, assigning as a reason it was unbecoming men of culture. The dwarfs who sneer at, and would ostracize mechanical dentistry can congratulate themselves in having a noted and hoary antecedent, for this occurred in the dark ages. From this debris sprang men of superior talent who seconded by the community cultivated surgery for its own merits. They availed themselves of all the light obtainable from medical science and their deeds achieved a victory over their traducers. The man who uses his hands and instruments over a dental chair is fully as much a mechanic, as the man who goes into his laboratory and by the same agencies constructs a piece of artificial work for the mouth. The difference between the men is the science and will force possessed and exercised respectively by them. Away with the twaddle that operative is superior to mechanical dentistry.

We who have a proclivity for mechanical dentistry need have no fears for our love. The operative dentists par excellence have not yet succeeded in preventing or treating satisfactorily diseases of the teeth. If success had been attained what apology could there be for the "new departure?" Appearances indicate much dissatisfaction and want of confidence in gold as a filling material. This is an age of reflection deep and irresistable; either you must defend your dogmas before a bar of reason or see them antiquated. No galvanizing defunct forms; you can never shape facts to suit yourself.

A few years ago the majority of dentists smiled at the idea of vulcanite, so common, cheap, and easily manipulated a material ever taking precedence over the noble metals gold and platinum. They have all ceased smiling. Within the recollection of many, the accepted treatment of disease by physicians was copious in general blood letting and the exhibition of mercury to salivation; to-day such treatment is universally condemned and considered mal-practice and murder. Men have been tinkering the human teeth for ages and have succeeded only in somewhat changing the surface aud ameliorating matters. The millenium will dawn on us before the demand for artificial teeth ceases.

A man may be able to make a very artistic gold filling in a tooth and construct a beautiful and elaborate set of artificial teeth and still be a miserable dentist. The most noted case which occurs in the annals of surgery, of what manual dexterity can do in the absence of all other qualifications, is Frere Jacques, the lithotomist, who was profoundly ignorant of anatomy when he entered upon his career as an operator; his success was such as to excite the wonder of educated surgeons. He was finally induced to study anatomy, by which his mind was improved, and he also adopted better appliances than he had been using; his success was greater by so doing; his course was guided by scientific principles which always underlie successful and honorable distinction.

The foregoing character is referred to in order to stimulate us to a laudable ambition in our life-work, to go deeper in medical science. There is no department of medicine but that a dentist can utilize; there is no patent on the powers of the mind or science. The same general principles of anatomy, physiology, pathology, and therapeutics belong in common to the general medical practitioner, surgeon and dentist.

